# Enteric Fever

**Published:** 1881-05

**Authors:** 


### ENTERIC FEVER.

Dr. Bristowe considers the treatment of enteric fever under four heads: 1. Diet; 2. Medicine; 3. Alcohol; 4. Baths; and in concluding his paper says: "Let me state briefly the treatment to which I should like to be subjected if ever, unfortunately, I should become affected with enteric fever. I should like to be placed in a cool, well-ventilated room, and covered lightly with bedclothes; to have a skillful and attentive nurse to look after me; to be fed solely with cold milk, unless vomiting should demand the addition to the milk of medicine calculated to allay vomiting. If diarrhœa became troublesome, or ever there was much pain or tenderness in the cœcal rings and in the bowels, I should like to be treated, not with laxatives, but with opium, given either by the mouth or the rectum. If constipation were present, I should, excepting in the first week, like to have enemata only for its relief. In the event of intestinal hæmorrhage coming on, I should like to have ice to suck or ice-cold fluids to drink, cold compresses to the belly, and cold injections into the bowels; and, though I am skeptical as to their efficacy, I should still choose to have astringents, and more especially lead, given to me at short intervals. If perforation should take place, let me have large and repeated doses of opium. Stimulants I should prefer to be without early in the disease; later, however, and during convalescence, I should like to have them in moderation. As to the cold baths, I would rather not have them; but I would, nevertheless, leave it to my physician to exercise his discretion in the matter. I would leave it also for him to decide, according to circumstances, whether alcohol should be administered to me in large quantities. I should prefer not to be treated at a temperance hospital."

